# Mother's Own Milk and Its Relationship to Growth and Morbidity in a Population-based Cohort of Extremely Preterm Infants

**DOI:** 10.1097/MPG.0000000000003352

**Published:** 2021-11-10

**Authors:** Anna-My Lund, Magnus Domellöf, Aldina Pivodic, Ann Hellström, Elisabeth Stoltz Sjöström, Ingrid Hansen-Pupp

**Affiliations:** ∗ Division of Paediatrics, Department of Clinical Sciences, Lund, Lund University and Skåne University Hospital; † Division of Paediatrics, Department of Clinical Sciences, Umeå University; ‡ Section for Ophthalmology, Department of Clinical Neuroscience, Institute of Neuroscience and Physiology, Sahlgrenska Academy, University of Gothenburg; § Department of Food, Nutrition and Culinary Science, Umeå University, Sweden.

**Keywords:** bronchopulmonary dysplasia, donor milk, human milk, pasteurization, retinopathy of prematurity

## Abstract

**Objectives:**

The aim of the study was to evaluate the relationships between intake of mother's own milk (MOM), compared with intake of pasteurized donor milk (DM), and postnatal growth, incidence of retinopathy of prematurity (ROP) and bronchopulmonary dysplasia (BPD), in extremely preterm infants.

**Methods:**

Swedish population-based cohort of surviving extremely preterm infants born 2004 to 2007. Exposure to MOM and DM was investigated from birth until 32 weeks postmenstrual age (PMA) in 453 infants. Primary outcome variables were change in *z*-score (Δ) from birth to 32 weeks PMA for weight, length, and head circumference (HC). Secondary outcomes were incidence of ROP and BPD. Mixed models adjusting for confounders were used to investigate the association between exposures and outcomes.

**Results:**

Infants’ mean gestational age (GA) was 25.4 weeks. Unadjusted, MOM (per 10 mL · kg^−1^ · day^−1^) was associated with Δweight and ΔHC with beta estimates of 0.03 *z*-score units (95% CI, 0.02–0.04, *P* < 0.001) and 0.03 *z*-score units (95% CI, 0.01–0.05, *P* = 0.003), respectively. After adjustment for predefined confounders, the association remained significant for Δweight and ΔHC. A similar pattern was found between Δweight and each 10% increase of MOM. Unadjusted, a higher intake of MOM (mL · kg^−1^ · day^−1^) was significantly associated to a lower probability of any ROP and severe ROP; however, these associations did not remain in the adjusted analyses. No associations were found between MOM (mL · kg^−1^ · day^−1^) and BPD. Moreover, no associations were found between DM and growth or morbidity outcomes.

**Conclusions:**

An increased intake of MOM, as opposed to DM (and not formula feeding), was associated with improved postnatal weight gain and HC growth from birth until 32 weeks PMA in extremely preterm infants. Interventions aiming at increasing early intake of unpasteurized MOM for extremely preterm infants should be encouraged.

**What Is Known/What Is New**

**What Is Known**

- A predominant intake of mother's own milk, as opposed to pasteurized donor milk, is associated with improved postnatal growth in preterm infants.
- Some studies suggest that in preterm infants, a predominant intake of mother's own milk compared with a predominant intake of pasteurized donor milk may prevent neonatal morbidities, such as infections, retinopathy of prematurity, and bronchopulmonary dysplasia.

**What Is New**

- The first study performed in a large national population-based cohort of extremely preterm infants where the effects of unpasteurized mother's own milk and pasteurized donor milk on postnatal growth and morbidities has been evaluated.

The advancement of medical, nursing, and nutritional care of extremely preterm infants continues to improve the survival of these vulnerable infants (1–3). The incidence of neonatal morbidities, however, remains high (2–4). Despite improved nutritional regimes, postnatal growth restriction is still often observed among extremely preterm infants (5). Inadequate growth in the postnatal period has been associated with incidence and severity of neonatal morbidities, such as retinopathy of prematurity (ROP), bronchopulmonary dysplasia (BPD), and poor neurodevelopmental outcomes (6–8).

Postnatal growth is a complex and multifaceted process influenced by factors, such as degree of immaturity, ability to assimilate nutrients, the nutritional composition of feeds, and presence of neonatal morbidities. In spite of the improvement of parenteral nutrition (PN) solutions and infant formulas, it remains clear that human milk is the optimal nutrition for the preterm infant provided that it is fortified to meet nutritional requirements.

Unpasteurized mother's own milk (MOM) is recommended as the primary choice and pasteurized donor milk (DM) as the secondary alternative (9,10). Benefits observed with MOM, over DM, in the preterm infant population may be because of the fact that the macronutrient content of unfortified DM usually is lower than that of unfortified MOM, partly as most DM originates from mothers delivering term infants and the milk is usually expressed at a later stage of lactation (11,12). Furthermore, lipase is inactivated as a result of the pasteurization process of human milk and this has been shown to reduce the lipid absorption in preterm infants (13).

In the last decade, there has been an increase in studies reporting beneficial outcomes of postnatal growth and incidence of neonatal morbidities, for preterm infants receiving a greater proportion of MOM compared with DM (14–20). To further evaluate the effects of MOM and DM in relation to postnatal outcomes in a large cohort of exclusively extremely preterm infants, we studied a Swedish national population-based cohort of infants born before 27 completed weeks of gestation. We hypothesized that infants who had a greater intake of unpasteurized MOM compared with pasteurized DM would have a more beneficial growth and a lesser incidence of neonatal morbidities based on the rationale that the pasteurization process affects the biological properties, such as immunoglobulins, enzymes, and growth hormones, of human milk negatively (21,22).

## METHOD

### Study Population

This study constitutes a sub-cohort of the prospective national population-based *Extremely Preterm Infants in Sweden Study* (EXPRESS), which included all infants born before 27 completed weeks of gestation between 2004 and 2007 in Sweden (23). For a total of 602 infants who survived the first 24 hours of life, nutritional data was retrospectively retrieved from hospital records (24). In this sub-cohort, all infants surviving to at least 32 weeks postmenstrual age (PMA) (n = 515) and that had nutritional data available until at least 32 weeks PMA as well as data for the primary outcome, that is, any growth measurement at 32 weeks PMA, were included (n = 509). Infants with chromosomal anomalies or major malformations were excluded because of expected interference with growth (n = 35). Those were heart malformations (n = 29), intestinal malformations (n = 1), limb reduction malformations (n = 2), multiple malformations (n = 2), and chromosomal anomalies (n = 1). Infants who received any formula between birth and 32 weeks PMA were also excluded (n = 21). For the head circumference (HC) outcome, infants with hydrocephalus were excluded from the analyses. The final study cohort consisted of 453 infants.

### Ethics

Acquisition of data from the neonatal period was approved by the Regional Ethical Review Board, Lund University, Lund, Sweden (Dnr 42/2004 and Dnr 138–2008) to be performed without informed parental consent.

### Data Collection

Prenatal-, neonatal- and postnatal data, including morbidity, mortality, and growth measurements of weight, length, and HC, were prospectively collected for the first 180 days of hospitalization or until discharge or death (23,25). In the current sub-cohort, detailed data of received PN and enteral nutrition was collected daily for the first 28 postnatal days, and thereafter once weekly on standardized days, that is, postnatal days 35, 42, 49, and so forth until discharge, death or when data was rendered unobtainable (24). A comprehensive description of the nutritional regimes is presented in Supplemental Digital Content 1. Macronutrient intakes from parenteral sources and enteral human milk fortifiers were calculated based on the data provided by the manufacturers. The details regarding how macronutrient intakes from human milk sources were calculated are described in Supplemental Digital Content 1. Five out of 7 health care regions routinely analyzed the energy and macronutrient content of both MOM and DM by mid-infrared spectrophotometry, and these analyses guided the fortification practices (26). Human milk fortifiers were gradually introduced as full enteral volumes were reached (24,26). In this cohort, the precise amount of human milk fortifiers prescribed to MOM and DM, respectively are not known. Nor is it known how fortification practices were altered in cases of faltering growth or other clinical conditions. Usage of any product of human milk fortifiers (here defined as fortifiers including *both* energy and protein) was summarized as the proportion of days (%) with any human milk fortifier from birth through 32 weeks PMA. All retrieved data was registered in the computer-aided nutrition calculation program Nutrium (Nutrium AB, Umeå, Sweden). Potential nutrients from blood products were not included in the current analyses.

### Growth Measurements, Clinical Variables, and Definition of Morbidities

Weight, length, and HC measurements were converted to *z*-scores for the gestational age (GA) and sex-specific Fenton preterm infant growth reference (27). Small-for-gestational age at birth was defined as a birth weight corresponding to less than the 10th percentile on the Fenton preterm growth chart. Clinical variables (presented in Table 1) and definitions of morbidities are described in Supplemental Digital Content 2.

**TABLE 1:** Prenatal and neonatal characteristics and postnatal outcomes in the study cohort (n = 453)

	All infants, n = 453	MOM <20%, n = 50	MOM 20% to 80%, n = 100	MOM >80%, n = 303
Prenatal characteristics				
Preeclampsia	56/430 (13.0%)	6/46 (13.0%)	13/94 (13.8%)	37/290 (12.8%)
Any antenatal steroids	405/434 (93.3%)	47/49 (95.9%)	87/94 (92.6%)	271/291 (93.1%)
Vaginal delivery	191/450 (42.4%)	22/50 (44.0%)	38/100 (38.0%)	131/300 (43.7%)
Neonatal characteristics				
Sex (female)	204/453 (45.0%)	23/50 (46.0%)	43/100 (43.0%)	138/303 (45.5%)
Gestational age, weeks	25.4 (1.1)25.6 (22.1; 26.9)	25.2 (1.1)25.5 (23.3; 26.9)	25.4 (1.1)25.6 (23.3; 26.9)	25.4 (1.1)25.6 (22.1; 26.9)
Birth weight, g	775 (167)770 (348; 1315)	751 (143)718 (504; 970)	759 (169)758 (428; 1180)	785 (170)775 (348; 1315)
Birth weight *z*-score	0.1 (0.9)0.2 (−2.9; 2.3)	+0.0 (0.8)0.1 (−2.1; 1.7)	−0.0 (1.0)+0.0 (−2.7; 2.0)	0.1 (0.9)0.2 (−2.9; 2.3)
Birth length *z*-score	0.1 (0.9)0.2 (−3.9; 2.4)(n = 359)	+0.0 (0.9)−0.1 (−2.3; 2.0)(n = 41)	−0.1 (1.0)+0.0 (−3.9; 2.3)(n = 80)	0.2 (0.9)0.3 (−3.3; 2.3)(n = 238)
Birth head circumference *z*-score	0.3 (0.8)0.3 (−2.5; 2.3)(n = 382)	0.2 (0.8)0.2 (−1.6; 2.0)(n = 42)	0.1 (0.8)0.1 (−2.1; 1.9)(n = 79)	0.4 (0.8)0.5 (−2.5; 2.3)(n = 248)
Small-for-gestational age at birth	39/453 (8.6%)	2/50 (4.0%)	11/100 (11.0%)	26/303 (8.6%)
Postnatal outcomes				
Total days of mechanical ventilation postnatal weeks 1 to 10	14 (15)9 (0; 70)	16 (17)10 (0; 64)	14 (16)8 (0; 70)	14 (14)9 (0; 70)
Total days of steroid treatment postnatal weeks 1 to 10	4 (8)0 (0; 53)	5 (10)0 (0; 47)	5 (9)0 (0; 37)	3 (8)0 (0; 53)
Retinopathy of prematurity	329/449 (73.3%)	40/50 (80.0%)	69/100 (69.0%)	220/299 (73.6%)
Severe retinopathy of prematurity	161/449 (35.9%)	22/50 (44.0%)	38/100 (38.0%)	101/299 (33.8%)
Treatment retinopathy of prematurity	90/449 (20.0%)	12/50 (24.0%)	19/100 (19.0%)	59/299 (19.7%)
Bronchopulmonary dysplasia	349/440 (79.3%)	42/49 (85.7%)	70/95 (73.7%)	237/296 (80.1%)
Severe Bronchopulmonary dysplasia	126/440 (28.6%)	14/49 (28.6%)	29/95 (30.5%)	83/296 (28.0%)
Δweight 32 weeks	−1.2 (0.7)−1.2 (−3.1; 0.6)	−1.3 (0.6)−1.3 (−2.5; −0.0)	−1.2 (0.7)−1.2 (−3.1; 0.6)	−1.1 (0.7)−1.2 (−2.9; 0.6)
Δlength 32 weeks	−1.5 (0.7)−1.5 (−3.7; 1.1)(n = 275)	−1.7 (0.6)−1.4 (−3.2; −0.9)(n = 28)	−1.4 (0.8)−1.3 (−3.7; 0.3)(n = 50)	−1.5 (0.7)−1.5 (−3.7; 1.1)(n = 197)
Δhead circumference 32 weeks	−1.5 (0.9)−1.4 (−3.6; 0.6)(n = 303)	−1.4 (0.9)−1.6 (−2.9; 0.3)(n = 28)	−1.4 (0.9)−1.4 (−3.2; 0.5)(n = 59)	−1.5 (0.9)−1.4 (−3.6; 0.6)(n = 216)

Continuous variables are presented as mean (SD) and median (Min; Max). Categorical variables are presented as number of infants (%). Δ = change in anthropometry *z* score from birth until 32 weeks postmenstrual age.

### Exposure Variables

In Sweden, extremely preterm infants are offered pasteurized DM as a complement to MOM until at least 32 weeks PMA after which DM is replaced by preterm formula. Our objective was to study differences in outcome based on respective intakes of MOM and DM, hence the exposure period was defined as beginning at birth and ending at 32 weeks PMA. Intake of MOM and DM (mL · kg^−1^ · day^−1^) together with the proportion of MOM of total breast milk (MOM%), constituted the exposure variables. Due to the breast milk data being recorded with different time intervals, the mean area under the curve (AUC) was calculated as a proxy for the mean intake of MOM and DM from birth until 32 weeks PMA for each infant. For interpretation of univariable and multivariable models, these variables were rescaled into increments of 10 mL · kg^−1^ · day^−1^ or 10% by dividing the mean AUC by 10.

### Outcome Variables

The primary outcome variables were change in *z*-score (denoted as delta, Δ) from birth to 32 weeks PMA for weight, length, and HC. The secondary outcome variables were any and severe ROP, ROP treatment, and any and severe BPD. We chose to focus on these morbidity outcomes as they are diagnosed after the exposure period (ie, after 32 weeks PMA) as opposed to other neonatal morbidities, such as sepsis and necrotizing enterocolitis, which often occur before 32 weeks PMA.

### Statistical Analyses

The data was analyzed in IBM SPSS Statistics for Windows, version 26.0 (IBM Corp., Armonk, NY) and SAS software version 9.4 (SAS Institute Inc., Cary, NC). Descriptive data are presented by mean (SD) and median (Min; Max) for continuous variables, and number and percentage for categorical variables.

Associations between exposure and outcome variables were examined with univariable and multivariable linear mixed models for continuous outcomes and generalized linear mixed models with binomial distribution and logit link function for dichotomous outcomes. The assumptions of normal distribution and homogeneity of residual variance in linear mixed models were examined through diagnostic plots and found fulfilled.

The following confounders were identified for both growth and morbidity outcomes: GA at birth, respective birth anthropometry *z*-score (for all growth outcomes) and birth weight *z*-score (for all morbidity outcomes), days with mechanical ventilation, days with postnatal steroid treatment, proportion of the volume of PN of the total volume of nutritional intake from birth through 32 weeks PMA (hereafter referred to as PN%) and health care region. Health care region was analyzed as a random effect. The proportion of days with any human milk fortifier was identified as a mediator, and therefore not adjusted for in the models.

Statistically significant associations in univariable analyses were further analyzed in multivariable analyses. Adjustment for confounders was performed in 2 different models for each of the studied outcomes of which the second model was considered to be the main analysis. The first model included the nonnutritional confounders GA, birth anthropometry *z*-score/birth weight *z*-score, mechanical ventilation, postnatal steroid treatment, and health care region. In addition to these, the second model also included the confounder PN%, which in this cohort represents both an additional source of nutrition (to MOM and DM) and degree of neonatal morbidity.

For all analyses, the tests were 2-tailed and an alpha less than 0.05 was considered significant. Missing data was not imputed. Beta estimates and odds ratios are presented with 95% confidence intervals (CI).

## RESULTS

Prenatal- and neonatal characteristics as well as postnatal outcomes for infants surviving to at least 32 weeks PMA (n = 453) are presented in Table 1, both for the cohort as a whole and divided into groups (<20%, 20–80%, and >80%) based on MOM%. Overall, the mean (SD) change in *z*-scores from birth until 32 weeks PMA were −1.2 (0.7) for weight, −1.5 (0.7) for length, and −1.5 (0.9) for HC.

### Breast Milk and Nutrition

The average (mean AUC) of energy and macronutrients originating from enteral, parenteral, and total nutritional intakes, human milk intakes and PN%, as well as the proportion of days for which the infants received any human milk fortifier, from birth until 32 weeks PMA are presented in Table 2 (as a whole and divided into MOM% groups). A total of 87 out of 453 infants (19.2%) never received any human milk fortifier.

**TABLE 2:** Intake of human milk and nutrition from birth until 32 weeks postmenstrual age in the study cohort (n = 453)

	All infants, n = 453	MOM <20%, n = 50	MOM 20% to 80%, n = 100	MOM >80%, n = 303
Human milk and human milk fortifiers				
Total human milk (mL · kg^−1^ · day^−1^)	123 (33)130 (6; 208)	138 (25)141 (26; 208)	125 (32)130 (8; 171)	120 (34)127 (6; 178)
Mother's own milk (mL · kg^−1^ · day^−1^)	94 (49)109 (0; 174)	5 (8)0 (0; 33)	68 (36)63 (4; 151)	117 (34)124 (5; 174)
Proportion of mother's own milk of total human milk (%)	74.6 (30.6)90.3 (0.0; 100.0)	4.5 (6.3)0.0 (0.0; 19.3)	54.7 (17.2)56.6 (21.0; 80.0)	92.8 (4.8)93.5 (80.2; 100.0)
Donor milk (mL · kg^−1^ · day^−1^)	29 (46)4 (0; 208)	133 (26)134 (26; 208)	57 (31)53 (2; 121)	3 (5)2 (0; 32)
Proportion of days with any human milk fortifier (%)	40.2 (27.6)45.9 (0.0; 89.7)	51.6 (22.4)53.0 (0.0; 87.3)	40.7 (28.8)46.4 (0.0; 88.5)	38.1 (27.6)41.4 (0.0; 89.7)
Never received any human milk fortifier	87/453 (19.2%)	3/50 (6.0%)	22/100 (22.0%)	62/303 (20.5%)
Fluids				
Total fluid (mL · kg^−1^ · day^−1^)	164 (15)164 (119; 223)	168 (15)167 (133; 217)	167 (17)168 (119; 215)	163 (15)163 (127; 223)
Enteral fluids (mL · kg^−1^ · day^−1^)	125 (35)132 (4; 212)	139 (26)143 (26; 212)	127 (34)133 (5; 176)	122 (36)130 (4; 180)
Parenteral fluids (mL · kg^−1^ · day^−1^)	40 (29)32 (1; 168)	28 (19)25 (2; 107)	40 (28)33 (2; 133)	41 (31)34 (1; 168)
Proportion of parenteral fluids of total fluids (PN%)	27.2 (18.6)23.4 (1.8; 97.7)	19.8 (12.8)18.4 (2.3; 80.6)	27.2 (18.1)24.3 (2.8; 96.4)	28.4 (19.4)23.9 (1.8; 97.7)
Energy				
Total energy (kcal · kg^−1^ · day^−1^)	113 (14)114 (59; 153)	117 (12)115 (88; 153)	114 (14)115 (59; 139)	112 (14)113 (66; 147)
Enteral energy (kcal · kg^−1^ · day^−1^)	94 (27)101 (2; 151)	104 (21)105 (17; 151)	95 (27)100 (3; 134)	93 (28)99 (2; 142)
Parenteral energy (kcal · kg^−1^ · day^−1^)	19 (16)14 (0; 85)	13 (11)9 (0; 72)	19 (15)15 (1; 85)	20 (16)15 (1; 83)
Protein				
Total protein (g · kg^−1^ · day^−1^)	2.9 (0.4)2.9 (1.8; 4.3)	2.8 (0.4)2.8 (2.1; 3.8)	2.9 (0.4)2.9 (2.1; 4.1)	2.9 (0.4)2.9 (1.8; 4.3)
Enteral protein (g · kg^−1^ · day^−1^)	2.3 (0.7)2.4 (0.0; 4.2)	2.5 (0.6)2.5 (0.3; 3.8)	2.4 (0.7)2.4 (0.1; 3.9)	2.3 (0.8)2.4 (0; 4.2)
Parenteral protein (g · kg^−1^ · day^−1^)	0.6 (0.5)0.4 (0.0; 3.1)	0.4 (0.4)0.3 (0.0; 2.5)	0.6 (0.5)0.4 (0.0; 3.0)	0.6 (0.6)0.4 (0.0; 3.1)
Carbohydrates				
Total carbohydrates (g · kg^−1^ · day^−1^)	11.8 (1.1)11.7 (7.7; 16.5)	12.0 (1.1)11.8 (10.1; 15.8)	12.0 (1.1)11.9 (7.7; 15.4)	11.7 (1.0)11.6 (9.3; 16.5)
Enteral carbohydrates (g · kg^−1^ · day^−1^)	8.8 (2.5)9.4 (0.2; 15.3)	10.0 (1.9)10.1 (1.8; 15.3)	9.0 (2.5)9.5 (0.3; 13.0)	8.6 (2.6)9.1 (0.2; 13.3)
Parenteral carbohydrates (g · kg^−1^ · day^−1^)	3.0 (2.4)2.4 (0.0; 14.1)	2.0 (1.6)1.7 (0.0; 9.5)	3.0 (2.3)2.4 (0.1; 11.2)	3.1 (2.6)2.5 (0.1; 14.1)
Lipids				
Total lipids (g · kg^−1^ · day^−1^)	5.8 (1.2)5.9 (0.9; 8.6)	6.1 (1.0)6.0 (3.6; 8.4)	5.8 (1.3)6.0 (2.2; 8.1)	5.7 (1.2)5.8 (0.9; 8.6)
Enteral lipids (g · kg^−1^ · day^−1^)	5.3 (1.6)5.5 (0.1; 8.5)	5.8 (1.3)5.7 (0.9; 8.4)	5.3 (1.6)5.6 (0.1; 8.0)	5.2 (1.6)5.5 (0.1; 8.5)
Parenteral lipids (g · kg^−1^ · day^−1^)	0.5 (0.5)0.3 (0.0; 3.3)	0.3 (0.4)0.2 (0.0; 2.8)	0.5 (0.5)0.3 (0.0; 3.3)	0.5 (0.5)0.3 (0.0; 2.6)

Continuous variables are presented as mean (SD) and median (Min; Max). Categorical variables are presented as number of infants (%).

#### Mother's Own Milk (mL · kg^−1^ · day^−1^) and Postnatal Growth

**FIGURE 1:**
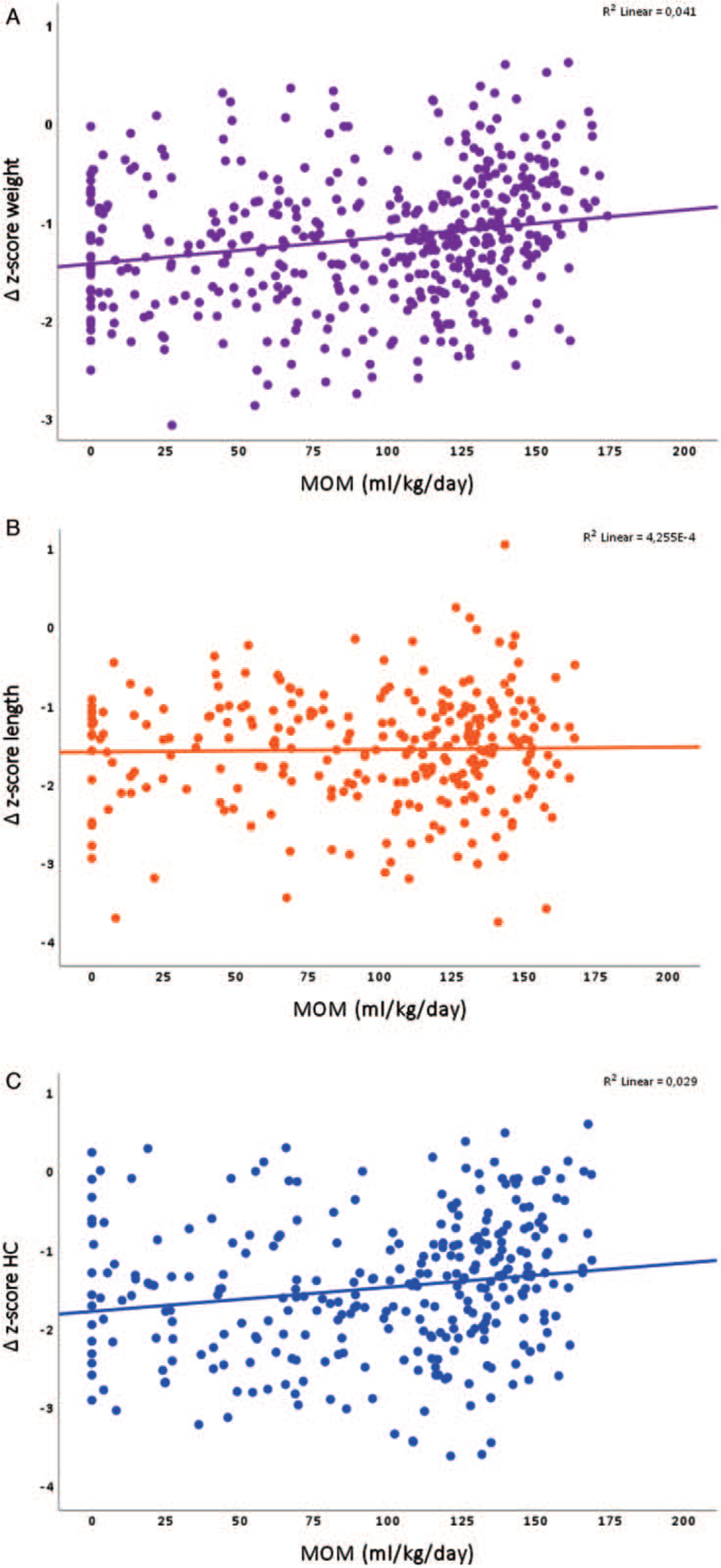
Univariable associations between mother's own milk and Δweight (A, n = 453), Δlength (B, n = 275) and Δhead circumference (HC, C, n = 303).

The univariable associations between MOM and the growth outcomes are depicted in Figure 1. There was a positive association between MOM and Δweight and ΔHC in the univariable models (Table 3). This association remained significant in multivariable models after adjustment for GA, respective birth anthropometry *z*-score, mechanical ventilation, postnatal steroid treatment, health care region, and PN% (multivariable models 1 and 2, Table 3). Each increase of 10 mL MOM per kg/day of the mean AUC estimated from birth through 32 weeks PMA would yield an increase of 0.02 (95% CI 0.01–0.03, *P* < 0.001) *z*-score units for Δweight and 0.02 (95% CI, +0.00–0.04, *P* = 0.049) *z*-score units for ΔHC from birth through 32 weeks PMA.

**TABLE 3:** Mother's own milk and its relationship to postnatal growth and neonatal morbidities

	OUTCOMES															
	Δweight, n = 453		Δlength, n = 275		ΔHC, n = 303		Any ROP,^∗^ n = 449		Severe ROP,^†^ n = 449		ROP treatment,^‡^ n = 449		Any BPD,^§^ n = 440		Severe BPD,^||^ n = 440	
Exposure	Beta (95% CI)	*P* value	Beta (95% CI)	*P* value	Beta (95% CI)	*P* value	OR (95% CI)	*P* value	OR (95% CI)	*P* value	OR (95% CI)	*P* value	OR (95% CI)	*P* value	OR (95% CI)	*P* value
Univariable model																
MOM (10 mL · kg^−1^ · day^−1^)	0.03 (0.02 to 0.04)	<0.001	+0.00 (−0.01–0.02)	0.733	0.03 (0.01 to 0.05)	0.003	0.93 (0.88–0.97)	0.001	0.93 (0.89–0.96)	<0.001	0.96 (0.91–1.00)	0.049	0.99 (0.95–1.04)	0.804	0.98 (0.94–1.02)	0.273
Multivariable model 1^¶^																
MOM (10 mL · kg^−1^ · day^−1^)	0.02 (0.02 to 0.03)	<0.001			0.03 (0.01 to 0.05)	<0.001	0.96 (0.91–1.01)	0.127	0.94 (0.90–0.99)	0.010	0.98 (0.93–1.03)	0.383				
Gestational age	0.01 (−0.03 to 0.05)	0.546			−0.00 (−0.09 to 0.09)	0.957	0.57 (0.42–0.77)	<0.001	0.64 (0.50–0.81)	<0.001	0.56 (0.42–0.73)	<0.001				
Birth anthropometry *z*-score^#^	−0.57 (−0.61 to −0.53)	<0.001			−0.63 (−0.72 to −0.54)	<0.001	0.94 (0.71–1.23)	0.628	0.74 (0.57–0.95)	0.019	0.68 (0.51–0.92)	0.011				
Mechanical ventilation	−0.00 (−0.01 to +0.00)	0.089			−0.01 (−0.02 to −0.00)	0.001	1.03 (1.00–1.06)	0.029	1.03 (1.01–1.05)	0.005	1.03 (1.01–1.05)	0.007				
Postnatal steroid treatment	−0.01 (−0.01 to −0.00)	0.003			−0.01 (−0.02 to +0.00)	0.054	1.02 (0.98–1.07)	0.319	1.02 (0.99–1.06)	0.129	1.02 (0.99–1.05)	0.166				
Multivariable model 2^¶^																
MOM (10 mL · kg^−1^ · day^−1^)	0.02 (0.01 to 0.03)	<0.001			0.02 (+0.00 to 0.04)	0.049	0.98 (0.93–1.03)	0.429	0.96 (0.92–1.01)	0.106	1.00 (0.94–1.06)	0.964				
Gestational age	0.02 (−0.03 to 0.06)	0.452			0.01 (−0.08 to 0.09)	0.905	0.56 (0.42–0.76)	<0.001	0.62 (0.49–0.79)	<0.001	0.54 (0.41–0.71)	<0.001				
Birth anthropometry *z*-score^#^	−0.57 (−0.61 to −0.53)	<0.001			−0.63 (−0.72 to −0.54)	<0.001	0.94 (0.71–1.24)	0.646	0.75 (0.58–0.97)	0.026	0.71 (0.53–0.95)	0.021				
Mechanical ventilation	−0.00 (−0.01 to +0.00)	0.211			−0.01 (−0.01 to −0.00)	0.019	1.02 (1.00–1.05)	0.106	1.02 (1.00–1.04)	0.023	1.02 (1.00–1.04)	0.022				
Postnatal steroid treatment	−0.01 (−0.01 to −0.00)	0.002			−0.01 (−0.02 to −0.00)	0.037	1.03 (0.98–1.08)	0.297	1.03 (0.99–1.06)	0.108	1.02 (0.99–1.05)	0.137				
Parenteral nutrition, %	−0.00 (−0.00 to +0.00)	0.158			−0.01 (−0.01 to −0.00)	<0.001	1.02 (1.00–1.04)	0.085	1.02 (1.00–1.03)	0.029	1.02 (1.00–1.03)	0.039				

The variable mother's own milk (MOM) represents the mean intake from birth until 32 weeks postmenstrual age and was rescaled into increments of 10 mL · kg^−1^ · day^−1^ for the analyses. Growth outcomes are presented as beta estimates with 95% confidence intervals (CI) and morbidity outcomes as odds ratios (OR) with 95% CI. Cells without numbers indicate that multivariable analysis was not performed. Δ = change in respective anthropometry *z*-score from birth until 32 weeks postmenstrual age; BPD = bronchopulmonary dysplasia; HC = head circumference; MOM = mother's own milk; ROP = retinopathy of prematurity;

∗ Any ROP was categorized into no ROP versus any stage of ROP.

† Severe ROP was categorized into no ROP or stages 1 to 2 versus ROP stages 3 to 5 and/or treatment of ROP (type 1 ROP).

‡ ROP treatment was categorized into no laser treatment versus laser treatment.

§ Any BPD was categorized into no BPD versus any stage of BPD.

|| Severe BPD was categorized into no BPD or supplemental oxygen ≤30% at 36 weeks postmenstrual age versus supplemental oxygen ≥30% at 36 weeks postmenstrual age.

¶ Health care region was analyzed as a random effect in the multivariable models.

# For the multivariable models assessing growth outcomes, the respective birth anthropometry *z*-score was used. For the multivariable models assessing morbidity outcomes, birth weight *z*-score was used.

#### Mother's Own Milk (mL · kg^−1^ · day^−1^) and Neonatal Morbidities

**FIGURE 2:**
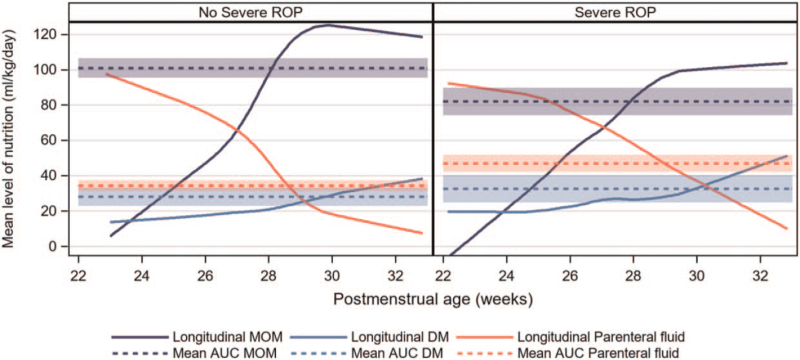
Longitudinal intakes (mL/kg/d) of mother's own milk, pasteurized donor milk, and parenteral nutrition according to postmenstrual age (weeks), in infants with (n = 161) and without (n = 288) severe retinopathy of prematurity are depicted by the filled lines. The calculations of the mean area under the curve (AUC) with 95% confidence intervals for MOM, DM, and PN intakes from birth through 32 weeks postmenstrual age are depicted by the dashed lines. DM = donor milk; MOM = mother's own milk; PN = parenteral nutrition.

A negative association was present between MOM and any ROP, severe ROP as well as ROP treatment in the univariable models, OR 0.93 (95% CI 0.88–0.97, *P* = 0.001); OR 0.93 (95% CI 0.89–0.96, *P* =  < 0.001); and OR 0.96 (95% CI 0.91–1.00, *P* = 0.049), respectively (Table 3). The association between MOM and severe ROP remained significant in multivariable models after adjustment for GA, birth weight *z*-score, mechanical ventilation, postnatal steroid treatment, and health care region (multivariable model 1, Table 3), OR 0.94 (95% CI 0.90–0.99, *P* = 0.010), but not after inclusion of PN% as a confounder (multivariable model 2, Table 3). Figure 2 depicts the respective longitudinal intakes (mL · kg^−1^ · day^−1^) of MOM, DM, and PN according to PMA for infants with and without severe ROP. There were no univariable associations between MOM and either any BPD or severe BPD (Table 3).

#### Proportion of Mother's Own Milk (%) in Relation to Postnatal Growth and Neonatal Morbidities

There was a positive association between MOM% and Δweight in the univariable models, but not for Δlength and ΔHC (Table, Supplemental Digital Content 3). The association between MOM% and Δweight remained significant in both multivariable models after adjustment for GA, birth weight *z*-score, mechanical ventilation, postnatal steroid treatment, health care region and PN% (multivariable model 2, Table, Supplemental Digital Content 3). Each increase of 10% MOM of the mean AUC estimated from birth through 32 weeks PMA would yield an increase of 0.03 (95% CI 0.02–0.04, *P* < 0.001) *z*-score units for Δweight from birth through 32 weeks PMA. There were no univariable associations between MOM% and any of the ROP or BPD outcomes.

#### Pasteurized Donor Milk (mL · kg^−1^ · day^−1^) in Relation to Postnatal Growth and Neonatal Morbidities

No univariable associations were present between DM and either Δweight, Δlength, ΔHC, any ROP, severe ROP, ROP treatment, any BPD or severe BPD (Table, Supplemental Digital Content 4).

## DISCUSSION

In a cohort of extremely preterm infants with detailed information regarding human milk and nutritional intakes, we evaluated the relationships between intake of MOM, and pasteurized DM, with postnatal growth and neonatal morbidity. In unadjusted models, increased intake of MOM (mL · kg^−1^ · day^−1^) improved weight gain and HC growth from birth until 32 weeks PMA and reduced the probability of any ROP and severe ROP development as well as treatment of ROP. After adjustment for predefined confounders, the associations between MOM (mL · kg^−1^ · day^−1^) and postnatal weight gain and HC growth remained significant. Similarly, the association between the proportional intake of MOM (MOM%) and postnatal weight gain remained significant in adjusted models. These results are in line with some previous studies that have shown beneficial growth outcomes for preterm infants fed predominantly MOM compared with DM (14–17).

We did not find any associations between DM (mL · kg^−1^ · day^−1^) and postnatal growth or neonatal morbidities. Notably, the infants in our cohort received significantly less DM than MOM and this may have affected the statistical power to determine relationships between DM and the outcomes.

Infants receiving predominantly pasteurized DM (ie, <20% MOM) from birth through 32 weeks PMA had a greater proportion of days with any human milk fortifier, a higher total intake of human milk as well as a slightly higher total energy intake than infants receiving predominantly MOM (ie, >80% MOM). The total protein intake was similar between the groups. This implies that other factors present in human milk, and not only the nutritional intake *per se*, may be of importance for infant growth.

Moreover, infants receiving predominantly pasteurized DM presented with a greater incidence of severe ROP (crude figures: 34% for infants receiving predominantly MOM and 44% for infants receiving predominantly DM). In a more contemporary cohort, we have previously shown that those infants with no ROP had a higher intake of MOM (mL · kg^−1^ · day^−1^) compared with infants with any ROP (20). Dani et al (28) recently presented that increasing MOM intake reduced the risk of ROP development in an Italian cohort of very preterm infants. In our cohort, however, the proportional intake of PN seemed to attenuate the relationship observed between MOM and severe ROP. Other studies have reported a lower incidence of ROP among preterm infants fed predominantly MOM compared with DM (18,19). Schanler et al (19) found that 5.6% of infants in the MOM group had ROP stage 3 compared with 19% in the DM group. Hence, the actual amount of MOM received seems to be of importance for ROP development.

We found no significant associations between MOM and BPD, which is in line with some other studies (15,16,19). The lack of an association may be because of the fact that 79% of the infants in our cohort had BPD, which makes it more difficult to discriminate a potential protective effect of MOM on BPD. Others have reported less severe BPD in the MOM group compared with the DM group (29) and a large population-based French study found a reduced risk of BPD for preterm infants fed unpasteurized compared with pasteurized MOM (30). Moreover, some literature reviews indicate that both partial and exclusive human milk feedings, that is, exclusive MOM or MOM supplemented with DM, as compared with formula feeding reduce the risk of BPD (31–33).

Strengths of our study include the large population-based sample, the detailed nutritional and growth data albeit its retrospective collection and that PN has been considered in the multivariable models. Limitations include differences in nutritional care between health care regions (although we adjusted for this in the multivariable models), the inability to determine potential differences in the actual amount of fortification used in MOM and DM, which is an important factor to consider when evaluating postnatal growth, the relatively old age of the data (infants born 2004–2007), and finally the observational design, which renders potential residual confounding in spite of our best efforts to identify and adjust for confounders.

The effect of the association observed between MOM and improved weight gain and HC growth in this cohort, although significant after adjustment for all identified confounders, was relatively small as indicated by the beta value, and thus the clinical significance of this finding could be questioned. It is, however, important to keep in mind that postnatal growth is a multifactorial phenomenon influenced by several factors, some of which are probably still unknown. Elucidating the respective effects of MOM compared with DM on postnatal growth would require a randomized clinical trial, which for ethical reasons is impossible.

## CONCLUSIONS

We found that MOM, as compared with DM, was associated with improved postnatal weight gain and HC growth in this national population-based cohort of extremely preterm infants. In regards to morbidity, we found associations between MOM and ROP, although not independent of other factors. No corresponding associations were observed for BPD. Notably, during the time of data collection, the nutritional care of extremely preterm infants in Sweden differed from the nutritional care received by infants born today. Thus, it seems relevant to further investigate the impact of MOM on both short- and long-term outcomes in a more contemporary large cohort of extremely preterm infants with equally detailed nutritional data. It is essential for neonatal health care professionals to include interventions that aim to increase the early intake of unpasteurized MOM in the care of extremely preterm infants.

## Supplementary Material

Supplemental Digital Content

## Supplementary Material

Supplemental Digital Content

## Supplementary Material

Supplemental Digital Content

## Supplementary Material

Supplemental Digital Content

## References

[1] van Goudoever JB. Nutrition for preterm infants: 75 years of history. *Ann Nutr Metab* 2018; 72:25–31. 29635225 10.1159/000487378 PMC6067646

[2] Norman M Hallberg B Abrahamsson T. Association between year of birth and 1-year survival among extremely preterm infants in Sweden during 2004–2007 and 2014–2016. *JAMA* 2019; 321:1188–1199. 30912837 10.1001/jama.2019.2021 PMC6439685

[3] Stoll BJ Hansen NI Bell EF. Eunice Kennedy Shriver National Institute of Child Health and Human Development Neonatal Research Network. Trends in care practices, morbidity, and mortality of extremely preterm neonates, 1993–2012. *JAMA* 2015; 314:1039–1051. 26348753 10.1001/jama.2015.10244 PMC4787615

[4] Stensvold HJ Klingenberg C Stoen R. Norwegian Neonatal Network. Neonatal morbidity and 1-year survival of extremely preterm infants. *Pediatrics* 2017; 139:e20161821. 28228499 10.1542/peds.2016-1821

[5] Figueras-Aloy J Palet-Trujols C Matas-Barceló I. Extrauterine growth restriction in very preterm infant: etiology, diagnosis, and 2-year follow-up. *Eur J Pediatr* 2020; 179:1469–1479. 32193657 10.1007/s00431-020-03628-1

[6] Hellström A Hård AL Engström E. Early weight gain predicts retinopathy in preterm infants: new, simple, efficient approach to screening. *Pediatrics* 2009; 123:e638–e645. 19289449 10.1542/peds.2008-2697

[7] Poindexter BB Martin CR. Impact of nutrition on bronchopulmonary dysplasia. *Clin Perinatol* 2015; 42:797–806. 26593079 10.1016/j.clp.2015.08.007

[8] Ehrenkranz RA. Nutrition, growth and clinical outcomes. *Nutritional Care of Preterm Infants* 2014; Basel: Karger, 11–26.

[9] Agostoni C Buonocore G Carnielli VP. ESPGHAN Committee on Nutrition. Enteral nutrient supply for preterm infants: commentary from the European Society of Paediatric Gastroenterology, Hepatology and Nutrition Committee on Nutrition. *J Pediatr Gastroenterol Nutr* 2010; 50:85–91. 19881390 10.1097/MPG.0b013e3181adaee0

[10] The American Academy of Pediatrics. Breastfeeding and the use of human milk. *Pediatrics* 2012; 129:e827–e841. 22371471 10.1542/peds.2011-3552

[11] Colaizy TT. Effects of milk banking procedures on nutritional and bioactive components of donor human milk. *Semin Perinatol* 2021; 45:151382. 33632557 10.1016/j.semperi.2020.151382

[12] de Halleux V Pieltain C Senterre T. Use of donor milk in the neonatal intensive care unit. *Semin Fetal Neonatal Med* 2017; 22:23–29. 27649995 10.1016/j.siny.2016.08.003

[13] Andersson Y Sävman K Bläckberg L. Pasteurization of mother's own milk reduces fat absorption and growth in preterm infants. *Acta Paediatr* 2007; 96:1445–1449. 17714541 10.1111/j.1651-2227.2007.00450.x

[14] Brownell EA Matson AP Smith KC. Dose-response relationship between donor human milk, mother's own milk, preterm formula, and neonatal growth outcomes. *J Pediatr Gastroenterol Nutr* 2018; 67:90–96. 29543698 10.1097/MPG.0000000000001959

[15] Montjaux-Régis N Cristini C Arnaud C. Improved growth of preterm infants receiving mother's own raw milk compared with pasteurized donor milk. *Acta Paediatr* 2011; 100:1548–1554. 21707744 10.1111/j.1651-2227.2011.02389.x

[16] de Halleux V Pieltain C Senterre T. Growth benefits of own mother's milk in preterm infants fed daily individualized fortified human milk. *Nutrients* 2019; 11:772. 10.3390/nu11040772 PMC6521225 30987136

[17] Soldateli B Parker M Melvin P. Human milk feeding and physical growth in very low-birth-weight infants: a multicenter study. *J Perinatol* 2020; 40:1246–1252. 32507858 10.1038/s41372-020-0705-2

[18] Dritsakou K Liosis G Valsami G. Improved outcomes of feeding low birth weight infants with predominantly raw human milk versus donor banked milk and formula. *J Matern Fetal Neonatal Med* 2016; 29:1131–1138. 25909500 10.3109/14767058.2015.1038232

[19] Schanler RJ Lau C Hurst NM. Randomized trial of donor human milk versus preterm formula as substitutes for mothers’ own milk in the feeding of extremely premature infants. *Pediatrics* 2005; 116:400–406. 16061595 10.1542/peds.2004-1974

[20] Lund AM Löfqvist C Pivodic A. Unpasteurised maternal breast milk is positively associated with growth outcomes in extremely preterm infants. *Acta Paediatr* 2020; 109:1138–1147. 31747093 10.1111/apa.15102 PMC9541184

[21] Hård AL Nilsson AK Lund AM. Review shows that donor milk does not promote the growth and development of preterm infants as well as maternal milk. *Acta Paediatr* 2019; 108:998–1007. 30565323 10.1111/apa.14702 PMC6520191

[22] Peila C Moro GE Bertino E. The Effect of Holder Pasteurization on Nutrients and Biologically-Active Components in Donor Human Milk: A Review. *Nutrients* 2016; 8:477. 10.3390/nu8080477 PMC4997390 27490567

[23] Fellman V Hellström-Westas L Norman M. One-year survival of extremely preterm infants after active perinatal care in Sweden. *JAMA* 2009; 301:2225–2233. 19491184 10.1001/jama.2009.771

[24] Stoltz Sjöström E Öhlund I Ahlsson F. Nutrient intakes independently affect growth in extremely preterm infants: results from a population-based study. *Acta Paediatr* 2013; 102:1067–1074. 23855971 10.1111/apa.12359

[25] The EXPRESS Group. Incidence of and risk factors for neonatal morbidity after active perinatal care: extremely preterm infants study in Sweden (EXPRESS). *Acta Paediatr* 2010; 99:978–992. 20456261 10.1111/j.1651-2227.2010.01846.x

[26] Stoltz Sjöström E Ohlund I Tornevi A. Intake and macronutrient content of human milk given to extremely preterm infants. *J Hum Lact* 2014; 30:442–449. 25117506 10.1177/0890334414546354

[27] Fenton TR Kim JH. A systematic review and meta-analysis to revise the Fenton growth chart for preterm infants. *BMC Pediatr* 2013; 13:59. 23601190 10.1186/1471-2431-13-59 PMC3637477

[28] Dani C Coviello C Panin F. Incidence and risk factors of retinopathy of prematurity in an Italian cohort of preterm infants. *Ital J Pediatr* 2021; 47:64. 33712037 10.1186/s13052-021-01011-w PMC7953747

[29] Ford SL Lohmann P Preidis GA. Improved feeding tolerance and growth are linked to increased gut microbial community diversity in very-low-birth-weight infants fed mother's own milk compared with donor breast milk. *Am J Clin Nutr* 2019; 109:1088–1097. 30982856 10.1093/ajcn/nqz006 PMC6462428

[30] Dicky O Ehlinger V Montjaux N. EPIPAGE 2 Nutrition Study Group, EPINUTRI Study Group. Policy of feeding very preterm infants with their mother's own fresh expressed milk was associated with a reduced risk of bronchopulmonary dysplasia. *Acta Paediatr* 2017; 106:755–762. 28128874 10.1111/apa.13757

[31] Villamor-Martínez E Pierro M Cavallaro G. Donor human milk protects against bronchopulmonary dysplasia: a systematic review and meta-analysis. *Nutrients* 2018; 10:238. 10.3390/nu10020238 PMC5852814 29461479

[32] Huang J Zhang L Tang J. Human milk as a protective factor for bronchopulmonary dysplasia: a systematic review and meta-analysis. *Arch Dis Child Fetal Neonatal Ed* 2019; 104:F128–F136. 29907614 10.1136/archdischild-2017-314205

[33] Villamor-Martínez E Pierro M Cavallaro G. Mother's own milk and bronchopulmonary dysplasia: a systematic review and meta-analysis. *Front Pediatr* 2019; 7:224. 31275904 10.3389/fped.2019.00224 PMC6593284

