# Supplementary material for: Mother's Own Milk and Its Relationship to Growth and Morbidity in a Population-based Cohort of Extremely Preterm Infants
Source: J Pediatr Gastroenterol Nutr. 2021 Nov 10;74(2):292–300. doi: 10.1097/MPG.0000000000003352 (PMC8788942; doi:10.1097/MPG.0000000000003352)
Supplement: Supplemental Digital Content [file jpga-74-292-s001.docx]

**Supplemental Digital Content 1**

**Nutritional regimes**

During the time when the EXPRESS cohort was conducted Sweden’s seven health care regions had different routines regarding the nutritional care of extremely preterm infants. Some hospitals used standardized prescriptions of parenteral glucose, amino acids, lipids, vitamins, minerals and trace elements whereas other hospitals individually prescribed parenteral solutions daily.

For enteral feedings mother’s own milk (MOM) was the primary choice. Pasteurized donor milk (DM) was available as a complement and was only given after parental consent. Five out of seven health care regions routinely analyzed the energy and macronutrient content of both MOM and DM by mid-infrared spectrophotometry (1). DM was pasteurized by Holder pasteurization (2). Macronutrient intakes from human milk sources were calculated based on the results from the human milk analyses for analyzed MOM and DM. For unanalyzed MOM and DM the following reference values, that are based on the analyzed human milk samples from the EXPRESS cohort, were used: early MOM (expressed prior to 28 days postpartum) was estimated to contain a content of 73 kcal, 1.83 g protein, 6.8 g carbohydrates and 3.97 g fat per 100 ml. Mature MOM (expressed after 28 days postpartum) and unanalyzed DM was estimated to contain a content of 70 kcal, 1.38 g protein, 7.0 g carbohydrates and 3.86 g fat per 100 ml.

When full enteral volumes were reached human milk fortifier was gradually introduced. However, the routines regarding usage and dosing, as well as the type of product chosen, of human milk fortifiers varied between health care regions as there were no formal guidelines for management of fortification during the time of the study.

**REFERENCES**

1. Stoltz Sjöström E, Ohlund I, Tornevi A, et al. Intake and macronutrient content of human milk given to extremely preterm infants. J Hum Lact 2014;30:442-9.

2. Hård AL, Nilsson AK, Lund AM, et al. Review shows that donor milk does not promote the growth and development of preterm infants as well as maternal milk. Acta Paediatr 2019;108:998-1007.
