# Supplementary material for: Mother's Own Milk and Its Relationship to Growth and Morbidity in a Population-based Cohort of Extremely Preterm Infants
Source: J Pediatr Gastroenterol Nutr. 2021 Nov 10;74(2):292–300. doi: 10.1097/MPG.0000000000003352 (PMC8788942; doi:10.1097/MPG.0000000000003352)
Supplement: Supplemental Digital Content [file jpga-74-292-s002.docx]

**Supplemental Digital Content 2**

**Clinical variables and definition of morbidities**

A detailed description of the clinical data collected has been described previously (1, 2). Briefly, retinopathy of prematurity (ROP) was defined according to the International Classification for Retinopathy of Prematurity (3). Severe ROP was defined as ROP stage 3 and/or treatment of ROP (Type 1 ROP) (4). Bronchopulmonary dysplasia (BPD) was defined as the need for supplemental oxygen at 36 weeks postmenstrual age (PMA), and further defined as severe BPD if the oxygen requirement was ≥ 30 % at 36 weeks PMA (2, 5). The need for mechanical ventilation, as well as administered postnatal systemic steroid treatment, was registered daily for the first ten postnatal weeks.

In the statistical analyses, we investigated ROP as three different dichotomous variables: any ROP (no ROP vs. any stage of ROP), severe ROP (no ROP or stages 1-2 vs. ROP stages 3-5 and/or treatment of ROP (Type 1 ROP)) and ROP treatment (no laser treatment vs. laser treatment). BPD was investigated as two different dichotomous variables: any BPD (no BPD vs. any stage of BPD) and severe BPD (no BPD or supplemental oxygen ≤ 30 % at 36 weeks PMA vs. supplemental oxygen ≥ 30 % at 36 weeks PMA).

**REFERENCES**

1. Fellman V, Hellström-Westas L, Norman M et al. One-year survival of extremely preterm infants after active perinatal care in Sweden. JAMA 2009;301:2225-33.

2. The EXPRESS Group. Incidence of and risk factors for neonatal morbidity after active perinatal care: extremely preterm infants study in Sweden (EXPRESS). Acta Paediatr 2010;99:978-92.

3. International Committee for the Classification of Retinopathy of Prematurity. The International Classification of Retinopathy of Prematurity revisited. Arch Ophthalmol 2005;123(7):991-9.

4. Early Treatment For Retinopathy Of Prematurity Cooperative Group. Revised indications for the treatment of retinopathy of prematurity: results of the early treatment for retinopathy of prematurity randomized trial. Arch Ophthalmol 2003;121(12):1684-94.

5. Jobe AH, Bancalari E. Bronchopulmonary Dysplasia. Am J Respir Crit Care Med 2001;163:1723-9.
