# Supplementary material for: Mother's Own Milk and Its Relationship to Growth and Morbidity in a Population-based Cohort of Extremely Preterm Infants
Source: J Pediatr Gastroenterol Nutr. 2021 Nov 10;74(2):292–300. doi: 10.1097/MPG.0000000000003352 (PMC8788942; doi:10.1097/MPG.0000000000003352)
Supplement: Supplemental Digital Content [file jpga-74-292-s003.docx]

**Supplemental Digital Content 3**

**Table. Proportion of mother’s own milk and its relationship to postnatal growth and neonatal morbidities.**

The variable proportion of mother’s own milk represents the mean intake from birth until 32 weeks postmenstrual age and was rescaled into increments of 10 % mother’s own milk/d for the analyses. Growth outcomes are presented as beta estimates with 95 % confidence intervals (CI) and morbidity outcomes as odds ratios (OR) with 95 % CI. Cells without numbers indicate that multivariable analysis was not performed.

| **EXPOSURE** | **OUTCOMES** | | | | | | | | | | | | | | | |
| --- | --- | --- | --- | --- | --- | --- | --- | --- | --- | --- | --- | --- | --- | --- | --- | --- |
|  | **∆weight**  **n=453** | | **∆length**  **n=275** | | **∆HC**  **n=303** | | **Any ROP^a^**  **n=449** | | **Severe ROP^b^**  **n=449** | | **ROP treatment^c^**  **n=449** | | **Any BPD^d^**  **n=440** | | **Severe BPD^e^**  **n=440** | |
|  | **Beta**  **(95 % CI)** | **P value** | **Beta**  **(95 % CI)** | **P value** | **Beta**  **(95 % CI)** | **P value** | **OR**  **(95 % CI)** | **P value** | **OR**  **(95 % CI)** | **P value** | **OR (**  **95 % CI)** | **P value** | **OR**  **(95 % CI)** | **P value** | **OR**  **(95 % CI)** | **P value** |
| **Univariable**  **model** |  |  |  |  |  |  |  |  |  |  |  |  |  |  |  |  |
| Mother’s own milk (10 %) | 0.02 (+0.00-0.04) | **0.0498** | −0.00 (−0.03-0.03) | 0.857 | −0.01 (−0.04-0.03) | 0.606 | 0.99 (0.93-1.06) | 0.847 | 0.96 (0.90-1.02) | 0.224 | 0.98 (0.91-1.06) | 0.593 | 1.00 (0.93-1.08) | 0.997 | 0.99 (0.93-1.06) | 0.748 |
| **Multivariable**  **model 1^f^** |  |  |  |  |  |  |  |  |  |  |  |  |  |  |  |  |
| Mother’s own milk  (10 %) | 0.02  (0.01-0.04) | **<0.001** |  |  |  |  |  |  |  |  |  |  |  |  |  |  |
| Gestational age | 0.01 (−0.03-0.05) | 0.585 |  |  |  |  |  |  |  |  |  |  |  |  |  |  |
| Birth anthropometry z-score^g^ | −0.57 (−0.61- −0.52) | **<0.001** |  |  |  |  |  |  |  |  |  |  |  |  |  |  |
| Mechanical ventilation | −0.00 (−0.01- −0.00) | **0.010** |  |  |  |  |  |  |  |  |  |  |  |  |  |  |
| Postnatal steroid treatment | −0.01 (−0.01- −0.00) | **0.008** |  |  |  |  |  |  |  |  |  |  |  |  |  |  |
| **Multivariable**  **model 2^f^** |  |  |  |  |  |  |  |  |  |  |  |  |  |  |  |  |
| Mother’s own milk  (10 %) | 0.03  (0.02-0.04) | **<0.001** |  |  |  |  |  |  |  |  |  |  |  |  |  |  |
| Gestational age | 0.02 (−0.02-0.06) | 0.209 |  |  |  |  |  |  |  |  |  |  |  |  |  |  |
| Birth anthropometry z-score^g^ | −0.57 (−0.62- −0.53) | **<0.001** |  |  |  |  |  |  |  |  |  |  |  |  |  |  |
| Mechanical ventilation | −0.00 (−0.01- +0.00) | 0.194 |  |  |  |  |  |  |  |  |  |  |  |  |  |  |
| Postnatal steroid treatment | −0.01 (−0.01- −0.00) | **0.003** |  |  |  |  |  |  |  |  |  |  |  |  |  |  |
| Parenteral nutrition (%) | −0.00 (−0.01- 0.00) | **<0.001** |  |  |  |  |  |  |  |  |  |  |  |  |  |  |
| ∆, change in respective anthropometry z-score from birth until 32 weeks postmenstrual age; HC, head circumference; ROP, retinopathy of prematurity; BPD, bronchopulmonary dysplasia.  ^a^ Any ROP was categorised into no ROP vs. any stage of ROP.  ^b^ Severe ROP was categorised into no ROP or stages 1-2 vs. ROP stages 3-5 and/or treatment of ROP (Type 1 ROP).  ^c^ ROP treatment was categorised into no laser treatment vs. laser treatment.  ^d^ Any BPD was categorised into no BPD vs. any stage of BPD.  ^e^ Severe BPD was categorised into no BPD or supplemental oxygen ≤ 30 % at 36 weeks postmenstrual age vs. supplemental oxygen ≥ 30 % at 36 weeks postmenstrual age.  ^f^ Health care region was analyzed as a random effect in the multivariable models.  ^g^ For the multivariable models assessing growth outcomes the respective birth anthropometry z-score was used. For the multivariable models assessing morbidity outcomes birth weight z-score was used. | | | | | | | | | | | | | | | | |
