# Supplementary material for: Mother's Own Milk and Its Relationship to Growth and Morbidity in a Population-based Cohort of Extremely Preterm Infants
Source: J Pediatr Gastroenterol Nutr. 2021 Nov 10;74(2):292–300. doi: 10.1097/MPG.0000000000003352 (PMC8788942; doi:10.1097/MPG.0000000000003352)
Supplement: Supplemental Digital Content [file jpga-74-292-s004.docx]

**Supplemental Digital Content 4**

**Table. Amount of donor milk and its relationship to postnatal growth and neonatal morbidities.**

The variable donor milk represents the mean intake from birth until 32 weeks postmenstrual age and was rescaled into increments of 10 ml/kg/d for the analyses. Growth outcomes are presented as beta estimates with 95 % confidence intervals (CI) and morbidity outcomes as odds ratios (OR) with 95 % CI.

| **EXPOSURE** | **OUTCOMES** | | | | | | | | | | | | | | | |
| --- | --- | --- | --- | --- | --- | --- | --- | --- | --- | --- | --- | --- | --- | --- | --- | --- |
|  | **∆weight**  **n=453** | | **∆length**  **n=275** | | **∆HC**  **n=303** | | **Any ROP^a^**  **n=449** | | **Severe ROP^b^**  **n=449** | | **ROP treatment^c^**  **n=449** | | **Any BPD^d^**  **n=440** | | **Severe BPD^e^**  **n=440** | |
|  | **Beta**  **(95 % CI)** | **P value** | **Beta**  **(95 % CI)** | **P value** | **Beta**  **(95 % CI)** | **P value** | **OR**  **(95 % CI)** | **P value** | **OR**  **(95 % CI)** | **P value** | **OR**  **(95 % CI)** | **P value** | **OR**  **(95 % CI)** | **P value** | **OR**  **(95 % CI)** | **P value** |
| **Univariable model** |  |  |  |  |  |  |  |  |  |  |  |  |  |  |  |  |
| Donor milk  (10 ml/kg/d) | −0.01 (−0.02-+0.00) | 0.183 | +0.00 (−0.01-0.02) | 0.673 | 0.02 (−0.01-0.04) | 0.197 | 1.01 (0.96-1.05) | 0.799 | 1.02 (0.98-1.06) | 0.340 | 1.00 (0.95-1.05) | 0.920 | 1.01 (0.96-1.07) | 0.664 | 1.01 (0.97-1.06) | 0.690 |
| ∆, change in respective anthropometry z-score from birth until 36 weeks postmenstrual age; HC, head circumference; ROP, retinopathy of prematurity; BPD, bronchopulmonary dysplasia.  ^a^ Any ROP was categorised into no ROP vs. any stage of ROP.  ^b^ Severe ROP was categorised into no ROP or stages 1-2 vs. ROP stages 3-5 and/or treatment of ROP (Type 1 ROP).  ^c^ ROP treatment was categorised into no laser treatment vs. laser treatment.  ^d^ Any BPD was categorised into no BPD vs. any stage of BPD.  ^e^ Severe BPD was categorised no BPD or supplemental oxygen ≤ 30 % at 36 weeks postmenstrual age vs. supplemental oxygen ≥ 30 % at 36 weeks postmenstrual age. | | | | | | | | | | | | | | | | |
